# The Bearing of Recent Biological Researches on the Practice of Medicine and Surgery

**Published:** 1892-03

**Authors:** 


### "THE BEARING OF RECENT BIOLOGICAL RESEARCHES ON THE PRACTICE OF MEDICINE AND SURGERY."

In a series of interesting articles under the above heading, published in the *London Lancet* from January 2d to February 6th, inclusive, Dr. G. Sims Woodhead has summarized very clearly the recent scientific researches and practical deductions of the leading biologists. No greater stimulus could be offered to scientific investigation than a study of the work accomplished by these patient, painstaking searchers after truth, and the wonderful, real, practical developments which their investigations have led to in all the branches of medicine and surgery. Many crooked ways have been made straight, and not a few dark medical corners illuminated. Some empirical methods of treatment have been firmly established upon rational bases and many relegated to oblivion. We have been taught why the "ring firing" practiced so assiduously for awhile, and persistently ridiculed a little later, is of real value and a scientifiic method of treating erysipelas. The following interesting quotation sets forth in detail the reasons why:

"It is objected by the opponents of the theory that the leucocytes (microphages) are always a little too late to catch the erysipelas streptococcus, the organism being found in a zone always a little beyond that in which we have any evidence of inflammation. Wood and Maxwell Ross showed that, although this was undoubtedly the case, the following inflammation appeared to exert a curative influence, in so far as it appeared to cut short the infective process. Metchnikoff* and Fehleisen, describing the ap-

*Virch. Archiv, Bd. cvii., 1887

pearances presented on microscopic examination, give first of all an outermost zone, where the erysipelas is spreading, but which to the naked eye appears almost normal, in which the micrococci, multiplying rapidly, completely fill the lymphatics. There is little or no evidence of inflammatory reaction. Passing inwards, however, we come to a zone in which inflammatory reaction has set in. The vessels are dilated, the fibrils of the connective tissue are swollen, and surrounding the small vessels and lying in the lymph spaces are numerous amœboid cells, leucocytes, or microphages, which, like their amœboid types, are acting the part of devouring cells, as many of them contain micrococci, sometimes in considerable numbers. The micrococci are usually so readily stained that they may easily be recognized in specially prepared specimens. Now comes the interesting point. In the next zone these smaller cells with their contents are in turn devoured by the larger amœboid cells—microphages, probably derived from the fixed connective cells, and each containing a single nucleus only. After this the organisms found in the tissues over which the disease has passed contain only dead and degenerated micrococci; so that, as Wood and Maxwell Ross say, 'It would thus seem that the wave of congestion, accompanied as it is by a diapedesis of the white blood corpuscles, has, in fact, cut short the course of the disease.' Arguing from this, they decided that if they could set up a certain amount of inflammation they could cut short the duration of the attack; in fact, that they might be able to localize it more or less completely according to the stage at which the disease came under treatment. They therefore decided to use iodine as a local irritant, applying it "in the form of a ring *slightly beyond that portion which is already affected.*" They say, 'It is naturally very important that inflammation should extend beyond the limits of the disease in order that it may act as a barrier to its further progress. It is, we think, very probably to the fact that the microbes have already penetrated in the tissues beyond the point where we have the visible signs of their action that we must ascribe the statement so frequently made, that although the treatment with iodine and with other irritants exerts a checking influence as a whole, yet at some point or other the affection still continues to spread. We may in these cases suppose that at some places our zone of irritation was not sufficiently in advance of the presence of the erysipelas cocci. The application of the irritant over the whole surface of the affected skin seems also, on the whole, undesirable, inasmuch as, in consequence of this, the dilatation of the vessels at the margins, which we have considered as the most important factor in the cure, must be interfered with by these being drained into the collateral vessels of the skin, and thus tending to diminish the intensity of the process in this region.' Basing their treatment on what had been observed, and bearing in mind the nature of the disease and of the spontaneous cure, they painted with iodine in the manner above described patients suffering from erysipelas, and in all cases where there was any chance of treating the disease the results obtained were most satisfactory, as the barrier of inflammation so set up appeared to serve its purpose most effectually. These observers, however, do not allow that the only effect of the inflammatory process is to bring up phagocytes to cope with the micrococci; they maintain, indeed, that there are two other very important factors to be taken into consideration. They hold that the dilatation of the vessels, with the increased flow of blood, may allow of the toxines produced by the microbe (which are said to have a paralyzing effect on the phagocytes, which are thus unable at first to ingest the active living microbes), whilst, in the second place, they think that the relatively increased supply of oxygen brought up by the increased quantity of blood may be sent on to 'the lymphatics, where the oxygen tension must naturally be very low. That this will favor the tissues rather than the microbes in their struggle is evident, when we consider that the streptococci of erysipelas in the animal body flourish best under anaërobic conditions, as is indicated by their strict limitation to the lymphatics.' But they say that 'the checking of the disease, by applying an irritant, such as iodine, in advance of it, indicates that the irritation to the cells, and the new set of conditions thereby induced, can alone cut short the affection.' Since the publication of the paper here referred to, Dr. Alex.

Miles, who assisted in carrying on the first series of experiments, has treated a large number of cases of erysipelas with marvellously successful results—results which, I am informed, will shortly be published."

The administration of antizymotic remedies in many of the infectious diseases has been shown to be either valueless or positively harmful; in others of decided and remarkable utility. The proper methods of disinfection much simplified, and its field of usefulness greatly extended. The greatest achievements, however, have been surgical. The preparation of operation field, sutures and dressings improved; the proper disinfection of operator and assistants evolved. The relative places of aseptic and antiseptic surgery have been accurately defined. The destruction of pathogenic bacteria by the leucocytes and connective tissue cells (phagocytosis), and the somewhat similar effect of the blood serum upon these micro-organisms, demonstrated by Metchnikoff and other biologists, explain the wonderful disease resisting powers of the animal, and especially of the human, organism.

The liver has been stripped of some of its supposed functions, and is no longer looked upon as the trusted sentinel standing guard over the blood, ready to arrest and finally execute the invading army of organic and chemical enemies which is constantly storming the citadels of life, but merely as an excretory (rather than secretory) gland.

That micro-organisms are the cause of infectious diseases seems to be conclusively proven; and many specific bacteria have been isolated, cultivated and shown by experiment upon man, as well as upon the lower animals, to produce specific diseases.

In conclusion I wish to quote the closing paragraphs of Dr. Woodhead's concluding article:

"This tissue resistance is so intimately bound up with the life history of the cell, with its powers of nutrition, and the facilities for excretion of effete matter, with its power of taking up foreign bodies of all kinds, pigment, bacteria, etc., and with its capacity for converting these into inert matter, and afterwards utilizing them for its own purposes, or of getting rid of them altogether. It has received special attention since the study of bacteria was recognized, and so much additional knowledge has already been obtained, that we may confidently look forward to a time when we shall be able to predicate what will be the effect of the introduction of tubercle bacilli, of leprosy bacilli, or of other pathogenic organisms, into different parts of the body, where they may be attacked by the different kinds of cells; we shall be able to point out what kinds of organisms are able to bring about degeneration of the various kinds of cells, and whether they do it directly through the action of their protoplasm, through the secretion of poisonous products, or through the presence of their dead protoplasm or of substances contained in it; we shall also be able to state which organisms hunt in couples, as it were—the one by its products preparing the way for the advance of the other,—so that where we find it difficult to get rid of both organisms we may be able to deprive one of its power of doing harm by destroying, banishing or rendering harmless the other; but, more important still, we shall be able to determine the conditions under which the various cells, tissues, and fluids of the body are best enabled to resist the advance and action of these organisms.

Although we are already well versed in the general laws of health, and, theoretically, are ready to meet every kind of disease by which we may be attacked, we have still not arrived at the conviction that, economically, the greater attention paid to the carrying out of mere preventive measures, such as better nutrition for the children of the poor, better conditions of hygiene, greater opportunities for exercise in the fresh air, would be a great saving to the State; whilst any expense that such improved measures might entail would be more than recouped by the great gain to the community as a whole, not merely through the better health and diminished death-rate in our present poverty stricken and overcrowded quarters, but also by the diminution in the number of centers from which disease may spread to those quarters where the conditions of life are much more favorable."

